# Medical Imaging in the Diagnosis of Schistosomiasis: A Review

**DOI:** 10.3390/pathogens10081058

**Published:** 2021-08-20

**Authors:** Andrea Cimini, Maria Ricci, Paola Elda Gigliotti, Luca Pugliese, Agostino Chiaravalloti, Roberta Danieli, Orazio Schillaci

**Affiliations:** 1 Department of Biomedicine and Prevention, University Tor Vergata, Via Cracovia 50, 00133 Rome, Italy; maria.ricci28@gmail.com (M.R.); paolaegigliotti@gmail.com (P.E.G.); l.pugliese88@gmail.com (L.P.); agostino.chiaravalloti@gmail.com (A.C.); orazio.schillaci@uniroma2.it (O.S.); 2 Department of Radiology, San Giovanni Calibita Fatebenefratelli Hospital, Via di Ponte di Quattro Capi 39, 00186 Rome, Italy; 3 Nuclear Medicine Section, Istituto di Ricovero e Cura a Carattere Scientifico (IRCCS) Neuromed, Via Atinense 18, 86077 Pozzilli, Italy; 4 Department of Human Sciences and Promotion of the Quality of Life, University San Raffaele, Via di Val Cannuta 247, 00166 Rome, Italy; roberta.danieli@uniroma5.it

**Keywords:** schistosomiasis, diagnostic imaging, helminths, X-rays, ultrasound, computed tomography, magnetic resonance imaging, positron emission tomography

## Abstract

Schistosomiasis is one of the most important parasitic diseases and it is endemic in tropical and subtropical areas. Clinical and laboratory data are fundamental for the diagnosis of schistosomiasis, but diagnostic imaging techniques such as x-rays, ultrasound (US), computed tomography (CT), magnetic resonance imaging (MRI), and positron emission tomography/computed tomography (PET/CT) may be helpful in the evaluation of disease severity and complications. In this context, the aim of this review is to explore the actual role of diagnostic imaging in the diagnosis of schistosomiasis, underlining advantages and drawbacks providing information about the utilization of diagnostic imaging techniques in this context. Furthermore, we aim to provide a useful guide regarding imaging features of schistosomiasis for radiology and nuclear medicine physicians of non-endemic countries: in fact, in the last years non-endemic countries have experienced important flows of migrants from endemic areas, therefore it is not uncommon to face cases of this disease in daily practice.

## 1. Introduction

Schistosomiasis (also known as bilharziasis) is a helminthic infection that usually occurs after direct contact with fresh water [1,2]. The World Health Organization (WHO) has included schistosomiasis in the list of neglected tropical diseases [3,4] (http://www.emro.who.int/health-topics/tropical-diseases (accessed on 6 August 2021)); moreover, it is considered as one of the most important parasitic diseases related to poverty in developing countries, being the second most common tropical disease after malaria [4].

Schistosomiasis is endemic in the tropical and subtropical areas (where it amounts to about 240 million cases), mostly in Sub-Saharan Africa [2,3,4], Latin America, Asia, although current evidence places it more and more in low or non-endemic areas, as Europe or North America, imported by returning travelers or migrants [3,5].

There are six species of Schistosoma [2,4], the three most associated with human disease are *Schistosoma haematobium* (*S. haematobium*), *Schistosoma mansoni* (*S. mansoni*) [2], and *Schistosoma japonicum* (*S. japonicum*) [3]. *S. haematobium* usually causes a urogenital disease, it is also related to bladder cancer and is endemic in Africa and Eastern Mediterranean. *S. mansoni* and *S. japonicum* localize mostly in the hepato-intestinal tract and are endemic respectively in Africa, Eastern Mediterranean, Caribbean, South America (mansoni), and South-East Asian and Western Pacific Region (japonicum) [4,6].

Schistosomiasis is caused by trematodes of the genus Schistosoma, that can be found in the fresh water of endemic areas. The parasite life cycle involves an intermediate host and an ultimate host. The cercariae, infecting free-swimming larvae, can actively penetrate the human skin: after that, they transform into schistosomula which travel within the venous blood vessels reaching the heart and lungs; subsequently, they come to the portal and venous intra-hepatic circulation, where they mature into the adult form, in two separate sexes (it is important to underline that sex is determined in fertilized eggs). The mature form may withstand the host immune response through various strategies, including the productions of molecules recognized as self-antigens [7]. The adult male-female couples migrate through circulation into different organs depending on the species, where the female begins to produce eggs (up to several hundred per day), for many years (up to 20): *S. japonicum* worms migrate to the inferior mesenteric and superior hemorrhoidal veins, *S. mansoni* worms migrate to the superior mesenteric vein, and *S. haematobium* adult worms migrate to the vesical plexus and veins draining the ureters, bladders, and other pelvic organs. Eggs are highly immunogenic, so they trigger an important inflammation reaction which causes tissues damage. They also produce lytic enzymes, which destroy tissues, and so they leave the vessels and pour into the bowel or bladder. Schistosome eggs are excreted by urine and feces, reaching the water. At this moment, the egg releases miracidia (the initial larval stage) which penetrates the intermediate host (aquatic snail) and undergoes several cycles of asexual reproduction, up to reaching the cercaria stage. Hence, cercaria is released by snail into the water, infecting again the final host [1,2,4,8].

There are many types of clinical manifestation of the disease, that is usually divided into acute and chronic form; the acute form (also called Katayama syndrome) is caused by immediate or delayed hypersensitivity reactions to the immature worm migrating in hepatic and other vessels [2], arising 14–84 days after the infection [9]. Symptoms are fever, malaise, nausea, headache, diarrhea [5,10,11], wheeze, eosinophilia [2], and transient pulmonary infiltrates. This form may be asymptomatic or show mild symptoms [11]. Acute form can be self-limiting or become chronic and the intensity of the symptoms depends on the parasitic load and patient sensitivity; if it gets worse, it means that egg deposition has begun [4,11]. The chronic form may occur months to years after infection and it is caused by egg deposition, which induces tissue damage by the production of lytic enzymes and the establishment of an important T-cell-mediated immune response and delayed-type hypersensitivity reaction, with the production of granulomas and finally tissues fibrosis [12]. Based on the reports of the literature, the most involved organs by chronic schistosomiasis are:

- Bladder (urogenital schistosomiasis), presenting hematuria, glomerulopathies with nephrotic syndrome; urogenital schistosomiasis is one of the major causes of hematuria in tropical travelers and *S. haematobium* is the most frequent cause of this chronic form [4,13].
- Small intestine-colon, presenting chronic diarrhea (usually caused by *S. mansoni* and *S. japonicum*) [14,15].
- Hepatosplenic system with abdominal pain and hepatomegaly; hepatosplenic schistosomiasis is frequently caused by *S. mansoni* and *S. japonicum* [16].
- Lungs, where it causes fibrosis and arteriolar damage [6].
- Brain and spinal cord, with granuloma deposition and development of neurological disorders [11,17,18].
- Breast, genitals, eyes, skin [4].

Chronic infection, if not diagnosed, can lead to high morbidity and mortality, with the onset of unsolvable complications, such as renal failure, hepatic cirrhosis, portal hypertension, ascites, pulmonary hypertension, cor pulmonale, bladder carcinoma, and neurological complications, [2,3,11]. However, it is important to mention that severe disease occurs in a relatively small percentage of infected subjects (~9% of patients with schistosomiasis worldwide) [19].

Currently, the gold standard therapy is praziquantel, an anthelmintic drug that acts only against adult worms (and not against juvenile forms) and does not help against organ damage [2,20]. In non-endemic countries diagnosis of schistosomiasis may be difficult, since it can be misdiagnosed because of the unspecific clinical signs and a low index of suspicion [21], and loss of sensitivity of the direct diagnostic techniques (microscopy) [3,22]. Schistosomiasis should be suspected in travelers or immigrants coming from the endemic areas, with positive anamnesis for contact with fresh or stagnant water [21]. The diagnosis is based on epidemiologic data, clinical presentation, eosinophilia, and direct/indirect laboratory methods [1]. As regards laboratory evaluation, an important technique is represented by the microscopic examination of stools (for *S. japonicum* and *S. mansoni*) and filtered urines (for *S. haematobium*) to find the eggs with the Kato-Katz technique, since egg excretion is considered a marker of active infection [22]: this is the most used method in endemic countries due to its rapidity and cost-effectiveness (even if it has a variable sensitivity) and it is also recommended by WHO in case of intestinal schistosomiasis [23]. Laboratory evaluation also comprises serology (to find serum antibodies), which is non-specific because it cannot differentiate between active infection and former one [2,8], molecular assays such as polymerase chain reaction (PCR) for circulating DNA of the parasite in blood, stool, urine, saliva, tissues, which is proved to be a sensitive and specific method even in the non-endemic countries [24] or immunodiagnostic assays such as enzyme-linked immunosorbent assay (ELISA) for the detection of schistosomulae antigens (which helps the diagnosis in the early infection) [22] and of eggs antigens in blood (which is the standard screening method). Moreover, another important tool for the diagnosis is represented by tissue biopsy, especially for intestinal (*S. japonicum* and *S. mansoni*) and urinary schistosomiasis (*S. haematobium*) [1].

In this context, diagnostic imaging techniques may be useful in case of suspected diagnosis of schistosomiasis, in the staging of the disease, in the evaluation of disease severity and complications, and in follow-up after treatment [4] (it is also important to remember that the definitive diagnosis will depend on the observation of parasite eggs in tissues, feces or urine); furthermore, the use of imaging techniques such as X-rays, ultrasound (US), computed tomography (CT), magnetic resonance imaging (MRI), or positron emission tomography/computed tomography (PET/CT) allows a better comprehension of several aspects related to the pathophysiology of schistosomiasis. This review aims to explore the actual role of diagnostic imaging in the diagnosis of schistosomiasis, based on the reports in the literature. Furthermore, we aim to provide a useful guide for radiology and nuclear medicine physicians of non-endemic countries, that should be aware of imaging features related to schistosomiasis, especially in suspicious cases of this disease, such as in case of travelers or migrants from endemic areas (it is important to underline that in the last years non-endemic areas such as Europe has experienced important flows of migrants from endemic countries [10] and therefore, it is not unusual for radiology and nuclear medicine physicians to face cases of patients with schistosomiasis in their daily practice). Finally, we aim to underline the advantages and drawbacks of diagnostic imaging techniques in the diagnosis of schistosomiasis, providing useful information about the utilization in this context.

## 2. X-rays, Ultrasound, and Computed Tomography Findings

X-rays, US, and CT are very useful tools for the assessment of the disease severity: there are several reports described in literature concerning the utilization of these imaging modalities in the diagnosis of schistosomiasis, with important morpho-pathological features that could be identified.

### 2.1. Pulmonary Schistosomiasis

**Figure 1:**
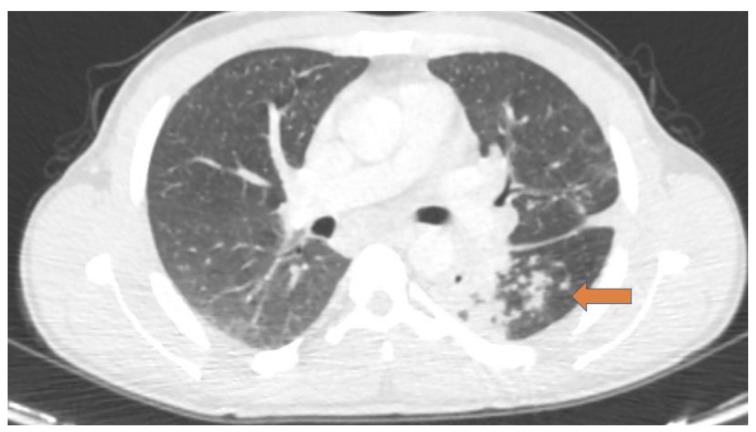
A case of Eritrean patient with chronic pulmonary schistosomiasis; axial computed tomography (CT) images showed multiple areas of consolidation in the left lung (orange arrow). A subsequent lung biopsy demonstrated parenchymal granulomas with associated schistosome eggs.

As regards pulmonary schistosomiasis, chest radiography may reveal nodular lesions, but it lacks sensitivity for subtle lesions [25]; CT scan may detect imaging signs which are missed or occult on chest radiography and it shows different alterations for each stage of the disease: typical findings of acute inflammation such as areas with ground-glass opacifications, micronodules, or reticular-nodular patterns are reported in acute pulmonary schistosomiasis (as part of Katayama syndrome, commonly seen in travelers in endemic areas following 3–8 weeks after the parasite entry) [4]. As regards chronic diseases (usually seen in migrants from endemic areas), radiological patterns of granulomas, arterioles, pulmonary fibrosis, or pulmonary hypertension, are frequently documented (these findings are related to the intense inflammatory reaction caused by egg deposition) [4,26]. Figure 1 shows a case of chronic pulmonary schistosomiasis evaluated with CT.

An interesting work from Gobbi et al. suggested the presence of a further stage of the lung disease, occurring at the beginning of chronic disease and called “early chronic disease”: in this intermediate phase, CT findings of acute disease such as ground glass lesions could be seen; authors hypothesized that in this phase (as the authors highlighted in their work, this phase may be defined radiologically but not clinically) female worms transit through the pulmonary parenchyma, laying their eggs in the lung [10]. The same authors reported the case of chronic pulmonary schistosomiasis treated with praziquantel, evaluating the response to treatment with CT scan [10].

### 2.2. Hepatointestinal Schistosomiasis

Granulomas and fibrosis (caused by egg deposition) are the main features of chronic hepatosplenic schistosomiasis and lead to portal hypertension [27]. US and CT may show important signs of portal hypertension such as periportal thickening, fibrosis, dilatation of splenic vein and splenomegaly with multiple splenic siderotic nodules (Gamma-Gandy bodies) [28], hence allowing a reliable assessment of disease severity and complications. US is generally the first-line imaging examination (due to its cost-effectiveness), showing peri-portal fibrosis (the essential lesion, as stated in the WHO practical guide for the assessment of schistosomiasis-related morbidity [https://apps.who.int/iris/handle/10665/66535 (accessed on 6 August 2021)]) and important findings such as left liver lobe hypertrophy and atrophy of right lobe and gallbladder wall thickening as well. Moreover, transient elastography may be used to evaluate liver stiffness for assessment of liver fibrosis in patients with advanced schistosomiasis [29]. It is also important to mention a possible imaging pattern of chronic schistosomiasis in the liver caused by *S. japonicum* and visible on US, consisting of “mosaics” with echogenic septa [16].

As concerns CT scan in the evaluation of chronic hepatosplenic schistosomiasis, this imaging technique may demonstrate important findings in the liver such as concentric enhancement of portal venous system structures, because of periportal fibrosis and inflammation, as clearly reported in an interesting case of a Brazilian woman with *S. mansoni* infection and hepatosplenic involvement that presented portal hypertension and esophageal varices. In addition, the authors described in this patient an associated wall-thickening of the gallbladder as a consequence of granulomatous inflammation [30].

An interesting CT pattern with calcifications was reported in *S. japonicum* infection by Araki et al. consisting in septal and capsular calcifications in the liver; moreover, the same authors found a concomitant hepatocellular carcinoma in 6 of 17 patients with hepatic schistosomiasis, suggesting a possible correlation between these two conditions [31].

As regards acute hepatosplenic schistosomiasis, this form is frequent among people exposed for the first time to the parasite (such as tourists in endemic areas): ultrasonographic features of the disease are represented by the enlargement of subdiaphragmatic lymph nodes and hepatosplenomegaly [32]. In this context, it is important to mention an interesting work by Cesmeli and collaborators [33], in which the authors reported the case of 27-year-old Caucasian female with a history of recent travel to Africa. The patient had a diagnosis of acute schistosomiasis with hepatic involvement: US showed several hypo-echoic nodules in the liver; a subsequent CT scan showed multiple hypodense lesions, with a complete disappearance of them after treatment with praziquantel.

As regards intestinal schistosomiasis, there are a few reports in the literature (evaluated mainly with CT scans) in which bowel wall-thickening and calcifications (due mainly to calcification of eggs deposited in submucosa and subserosa [34]) are the most frequent findings described. An interesting study from Shen et al. evaluated 42 patients with chronic schistosomiasis due to *S. japonicum* infection. In 21 of 42 patients, intestinal involvement was detected through CT scan, revealing wall thickening and calcifications in descending colon, sigmoid colon, and rectum: biopsies revealed fibrosis and calcified eggs in these sites [35].

The same findings were reported in a study from Valluru et al. as well, in which 50 patients with schistosomal associated appendicitis were evaluated with CT scan: these subjects presented mainly appendiceal wall-thickening and calcifications along colon wall in multiple sites, especially in the sigmoid colon and rectum [36].

A rare case of the polypoid lesion due to intestinal schistosomiasis was reported in 2020 by D’Souza et al. in a patient with a large bowel obstruction. CT scan demonstrated a cecal nodular wall-thickening and after a right hemicolectomy, final histopathological examination showed fibrosis and calcified eggs [37].

### 2.3. Genitourinary Schistosomiasis

*S. haematobium* infection is the most frequent cause of bladder wall calcification in endemic zones [38]. The degree of calcification depends on the number of calcified eggs in the bladder wall: therefore, late-stage chronic schistosomiasis leads to extensive bladder calcifications [39]. US is less sensitive than X-rays and CT in the detection of urinary schistosomiasis [40]; a typical pathognomonic pattern of chronic schistosomiasis may be shown with a pelvic radiograph, in which the image of a calcified bladder may resemble a fetal head in the pelvis [39]. There are also others described in the literature, represented by granular, linear, or irregular calcification, easily detectable with CT scan [39].

Moreover, squamous cell bladder cancer is an important sequela of chronic urinary schistosomiasis [41] and the extension of the malignancy could be assessed through contrast-enhanced CT [42].

CT is also an important tool for the evaluation of ureteral obstructions due to schistosoma infection: an interesting report from Lorca et al. described the case of a 34-year old Spanish female, with a previous diagnosis of *S. haematobium* infection after a trip to Myanmar, treated with praziquantel; after two years, uro-CT images detected left hydronephrosis due to a wall-thickening of the left distal ureter: postoperative pathological examination revealed the granulomatous reaction to schistosome eggs deposited in the left ureteral wall [43]. A similar case was reported by Pal and collaborators, in which a right hydronephrosis caused by *S. haematobium* infection in 41-year-old male (migrated from Zimbabwe to the United Kingdom 20 years before) was detected through CT imaging [44].

As regards genital schistosomiasis, male organs are frequently involved along with bladder (in case of *S.haematobium*) or intestine infection (in case of *S.mansoni*) [45]. Cozzi et al. underlined the usefulness of US in the evaluation of male genital schistosomiasis, especially in the detection of prostate alterations (such as calcified areas or thinned seminal vesicles walls) [46]. In addition, this imaging technique may be useful in the identification of testicle involvement (revealing edema or parenchymal alterations) [46]. Female genital schistosomiasis is considered an important threat to reproductive sexual health in endemic areas [47]: US may be useful to identify uterus, tube, or ovary involvement, although specific imaging alterations caused by schistosomiasis have not been highlighted yet (often these abnormalities may mimic genital tuberculosis) [46].

### 2.4. Central Nervous System Schistosomiasis

As regards schistosomiasis of the central nervous system, manifestations are related to the inflammatory reaction to eggs deposition in the brain and spinal cord [48]. *S. Japonicum* infection is the most frequent cause of cerebral schistosomiasis [48], whereas *S. haematobium* and *S. mansoni* involve the spinal cord [4]. CT may highlight interesting imaging findings especially for cerebral schistosomiasis, such as areas of edema or brain calcifications [49]. Moreover, Vale and collaborators reported two interesting cases of cerebral schistosomiasis due to *S. mansoni* infection, the first characterized by nodular and contrast-enhancing lesions in the left temporal lobe, pons, and thalamus: a final pathological examination of these lesions revealed multiple granulomas with schistosome eggs; in the second case, CT images showed a large hypodense area in the right parieto-occipital region, consisting in multiple necrotic granulomas caused by the deposition of schistosome eggs [50].

## 3. Magnetic Resonance Imaging Findings

To the best of our knowledge, there are a few reports in the literature regarding the utilization of MRI in the diagnosis of schistosomiasis: these reports regard mainly schistosomiasis of the central nervous system, hepatic, and genital schistosomiasis.

Concerning schistosomiasis of the central nervous system, Lu et al. presented an interesting work including seven patients with cerebral schistosomiasis lesions, consisting in granulomatous reactions around schistosome eggs. MRI scans showed nodular lesions in these patients, all located in circumscribed areas: the authors described some nodules hyperintense on T2 weight-images and hypointense on T1 weight-images, corresponding to necrotic granulomas; other nodular lesions were instead iso/hypointense on T2 weight-images and isointense on T1 weight-images, consisting in a productive stage of the granulomatous reaction. Moreover, T1 weight enhanced images were acquired in six patients, showing multiple enhancing nodules in all these cases [51]. In 2018, Suthiphosuwan et al. presented a particular case of a Canadian patient with a suspected brain tumor: contrast-enhanced MRI images revealed a mass-like lesion (characterized by multiple areas of nodular and linear enhancement) with perilesional edema. A biopsy of the lesion demonstrated a *S. mansoni* infection, with deposition of eggs. Moreover, subsequent questioning revealed that the patient visited Ghana 10 years before [52].

In this context, it is important to also mention the interesting study by Huang and collaborators that underlined the importance of diffusion-weighted imaging (DWI): the authors demonstrated that DWI examination with apparent diffusion coefficient (ADC) values of cerebral schistosomiasis lesions may be useful for a correct diagnosis [53].

MRI is a very important tool in the diagnosis of schistosomiasis of the spinal cord: this imaging technique shows typical findings of inflammatory myelopathies, such as edema of the spinal cord, cauda equina, and conus medullaris. Another important imaging finding is the thickening of nerve roots of cauda equina associated with a heterogenous contrast enhancement [54].

As regards hepatic schistosomiasis, Bezerra and collaborators demonstrated the usefulness of MRI in the differential diagnosis between chronic hepatic schistosomiasis and cirrhosis [55]: it is important to highlight that radiological feature of chronic hepatic schistosomiasis such as peripheral portal fibrosis, splenic siderotic nodules, hepatomegaly, splenomegaly, heterogeneity of liver parenchyma, and signs of portal hypertension are easily detectable on MRI [4,55]. Moreover, MRI may provide a clear picture of vessels, helping also in the planning of possible surgical approaches to portal hypertension [56]. As mentioned before, septal and capsular calcifications in the liver is a typical pattern reported in *S. japonicum* infection [31]: one limitation of MRI in the diagnosis of schistosomiasis is the difficulty to identify calcifications in the hepatic parenchyma, as demonstrated in a report from Bilgin et al. [57]. On the other hand, it is important to underline that this limitation is restricted to standard anatomical MRI; susceptibility-weighted imaging (SWI), an advanced MRI technique that has been successfully used to detect hepatic calcifications in another infectious disease (but has not been tested yet in schistosomiasis), may be useful for this purpose [58].

As concerns genital schistosomiasis, MRI is an excellent tool for the evaluation of chronic infection in prostate and seminal vesicles [4]. A case from Ferreira et al. showed the features on MRI of testicular involvement in male genital schistosomiasis, describing a nodular lesion hypointense in T2 weight images and characterized by intense enhancement after administration of paramagnetic contrast agent [59]. To the best of our knowledge there are a few reports regarding female genital schistosomiasis and MRI: however, it is important to underline that vulvar and perineal lesions or cases of Fallopian tube infected and blocked by *S. haematobium*, associated with tubo-ovarian abscess, may be seen [4,39].

## 4. PET/CT Findings

PET/CT is a diagnostic imaging technique that provides functional tomographic images, detecting the sites of increased metabolic activity [60]. ^18^F-fluorodeoxyglucose (^18^F-FDG) is a glucose analog (^18^F-FDG is taken up by the cells with the same transport mechanism as glucose) and it is one of the most used PET radiopharmaceuticals in clinical practice, especially for oncological purposes [61]. In addition, the use of ^18^F-FDG PET/CT in infections and inflammations is well documented in literature: guidelines drawn up by the European Association of Nuclear Medicine (EANM) and the Society of Nuclear Medicine and Molecular Imaging (SNMMI) suggest the utilization of ^18^F-FDG PET/CT imaging in acute and chronic infection, since high levels of glucose transporters (GLUT) are expressed in the cells involved in infections and inflammations [62].

**Figure 2:**
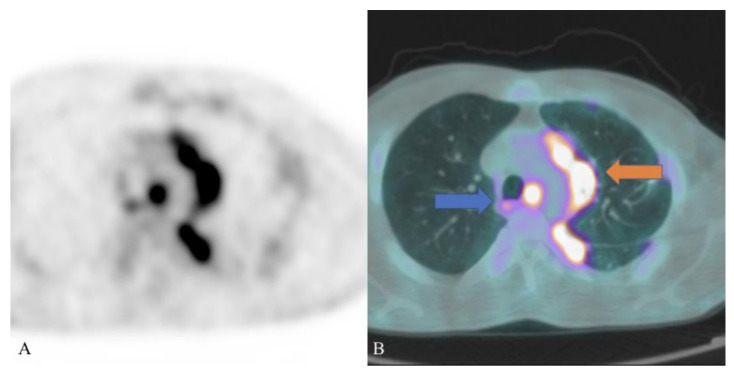
A case of pulmonary schistosomiasis evaluated with ^18^F-fluorodeoxyglucose (18F-FDG) positron emission tomography (PET, image (**A**)) and positron emission tomography/computed tomography (PET/CT, image (**B**)): ^18^F-FDG PET/CT axial images showed multiple hypermetabolic areas in the left lung (orange arrow, consisting in multiple granulomas caused by the deposition of schistosome eggs) and reactive lymph-nodes with high uptake of the radiopharmaceutical in the mediastinum (blue arrow).

There are a few cases reported in the literature regarding the utilization of ^18^F-FDG PET/CT in the diagnosis of schistosomiasis, suggesting a potential role of this imaging technique in the identification and localization of infection sites, helping also in the management of the disease: these cases (described below) demonstrated that schistosomiasis lesions are hypermetabolic on ^18^F-FDG PET/CT scan; moreover, schistosome eggs trapped in organs and tissues cause intense inflammatory reactions (including granulomas) [2] and therefore a high accumulation of the radiopharmaceutical in these sites. Figure 2 depicts a case of pulmonary schistosomiasis evaluated with ^18^F-FDG PET/CT.

As early as 1950 physiology experiments demonstrated that schistosomes are large consumers of glucose [63]: therefore ^18^F-FDG PET/CT imaging, as reported in a study with mice infected with *S. mansoni*, could be useful for the evaluation of worm burden in schistosomiasis infection [64]. Particularly noteworthy are also the results obtained in a study conducted by Lindner and collaborators, using mice infected with *S. mansoni*: the authors found a significantly higher uptake of ^18^F-FDG in the spleen of infected mice in comparison to the control group, suggesting a possible role of splenic uptake in the determination of infection grade [8].

Ye et al. presented the case of a 59-year-old man with two lesions in the liver and pancreas that showed increased uptake of the radiopharmaceutical, suspicious for metastatic pancreatic cancer: a subsequent postoperative biopsy revealed a granuloma of the liver due to an old hepatic schistosomiasis (with an old deposition of schistosome eggs) and chronic pancreatitis [65].

Altinyay et al. documented the case of 24-year-old Saudi man with lower back pain, lower-limb weakness, and urinary retention. MRI images of the spine were suggestive for longitudinal myelitis from T6 to T9, associated to a high count of white blood cells in the analysis of cerebrospinal fluid. The patient underwent a PET-CT scan with ^18^F-FDG that showed high uptake of the radiopharmaceutical in the spinal cord from T6 to T9, with a maximum standardized uptake value (SUVmax) 6.4. A subsequent laminectomy and spinal cord biopsy demonstrated schistosomiasis [66].

In 2019, Daghigh and collaborators published an interesting report of intestinal schistosomiasis, evaluated with ^18^F-FDG PET/CT imaging. The authors presented the case of a 36-year-old Eritrean man, who moved to Denmark three years before, with a history of abdominal pain, fever, nausea, and weight loss. Stool and urine analysis were positive for Schistosoma. The patient underwent a ^18^F-FDG PET/CT scan that showed pathologic hypermetabolism in abdominal lymph nodes, in the ileum, peritoneum, and omentum majus; furthermore, ascites characterized by moderate uptake of the radiopharmaceutical was documented [67].

In 2020, our research group documented a case of pulmonary schistosomiasis in a 32-year-old Ethiopian male patient, who recently moved to Italy: he presented dry cough, fever, weight loss and laboratory analysis demonstrated hypereosinofilia. PET/CT scan showed multiple hypermetabolic foci in the lung parenchyma with SUVmax 9.2 and multiple enlarged supradiaphragmatic lymphadenopathies characterized by high uptake of ^18^F-FDG (SUVmax 10.4 in the left hilar region). Lung biopsy was positive for granulomatosis associated with Schistosoma eggs [13].

As regards other PET tracers used in clinical practice, it is important to mention the report documented by Zhang et al. using ^18^F- sodium fluoride (^18^F-NaF), a bone-seeking radiopharmaceutical used for the detection of bone abnormalities (the uptake of this tracer reflects mainly bone remodeling and blood flow): the authors presented the case of a 77-year-old man with back pain; ^18^F-NaF PET/CT scan demonstrated high uptake of the tracer in the pelvic intestinal walls in correspondence with multiple calcifications, a sequela of a previous *S. japonicum* infection (the patient referred that he experienced the disease 30 years before) [68,69].

## 5. Discussion

The studies and cases reported in this review have underlined the usefulness and the importance of diagnostic imaging techniques in the diagnosis and the management of schistosomiasis: epidemiologic, clinical, and laboratory data are fundamental but diagnostic imaging may help in the evaluation of disease severity and complications; in addition, diagnostic imaging may be also useful for a better understanding of the pathophysiology of schistosomiasis.

As regards the utilization of X-rays in the diagnosis of schistosomiasis, generally this imaging technique is the first-line imaging examination for acute and chronic pulmonary schistosomiasis, despite it is less sensitive than CT to detect lesions [26]; in addition, pelvic radiograph has an important role in the diagnosis of urinary chronic schistosomiasis, detecting pathognomonic imaging patterns in case of calcified bladder [39].

The safety and the effectiveness of US for a rapid diagnosis are well-recognized in clinical practice [70]. As concerns the role of this imaging technique in the diagnosis of schistosomiasis, we have underlined its importance especially in case of chronic hepatic involvement: in the case of acute hepatic schistosomiasis, US may detect only non-specific findings such as hepatosplenomegaly, enlarged lymph nodes [71], and hypo-echoic nodules [33]. Regarding the chronic phase of hepatic schistosomiasis, US may easily evaluate the degree of periportal thickening, recognized as a prognostic indicator for gastrointestinal bleeding and survival [28]. Moreover, ultrasound transient elastography is considered an excellent imaging technique for the evaluation of heterogeneous liver fibrosis (with a better accuracy in comparison to CT) [72]. Finally, US is also an excellent and reliable tool for the evaluation of treatment response, assessing the eventual regression of hepatosplenic abnormalities [71]. Nevertheless, US efficiency is operator-dependent, representing the main limitation of this imaging technique in clinical practice [73].

There are several reports in the literature regarding the utilization of CT in the diagnosis of schistosomiasis, underlining its usefulness especially for the evaluation of pulmonary, intestinal, urinary, and central nervous system schistosomiasis; moreover, CT is an excellent imaging modality for the evaluation of calcifications in the liver (typical pattern with calcifications was described in *S. japonicum* infection) [31]. The main drawback of this imaging technique is represented by the exposure to high ionizing radiation doses [74]. Furthermore, in some cases iodinated-based contrast agents are used, with the possibility of adverse reactions after the intravenous administration; in addition, a screening of patients before the administration of contrast medium is mandatory: in particular, physicians should assess their renal function and should investigate previous reactions to iodinated contrast [75].

As we underlined in the cases reported in this review, MRI is an excellent imaging technique for the evaluation of central nervous system and genital schistosomiasis. Concerning central nervous system schistosomiasis, it is also important to highlight the possible role of MRI in the evaluation of treatment response, as demonstrated by Wen et al. in a case of a pediatric patient with neuroschistosomiasis [76]. This role was confirmed in an interesting case reported by Detzler et al., in which an Eritrean male patient showed a radiculopathy caused by *S. mansoni* infection: MRI was used for the evaluation after treatment (praziquantel and corticosteroids), showing regression of the radiculopathy [77]. Further studies are needed, but these cases highlight the promising role of MRI for this purpose.

The superiority of MRI in the diagnosis of hepatosplenic schistosomiasis in comparison to the other imaging modalities is well-recognized [78]; MRI may provide useful and accurate information regarding chronic hepatic schistosomiasis: in particular, contrast-enhanced MRI is useful for an accurate determination of the degree of periportal liver fibrosis or other features such as granulomatous inflammation, hepatomegaly, splenomegaly, large portal vein diameter, ascites, splenic siderotic nodules [4,55,79]. Moreover, as described before, MRI may allow a correct differential diagnosis between chronic hepatic schistosomiasis and other chronic conditions such as cirrhosis [55]. In comparison to CT, MRI images are obtained without using any ionizing radiation; on the other hand, MRI costs are much higher [28]. As regards future perspectives, new studies are highlighting the possible role of transverse relaxation time T2 as an imaging biomarker in the assessment of hepatic fibrogenesis, opening new possibilities in the monitoring of liver fibrosis in schistosomiasis [80].

Although the few cases described in the literature and the non-specific findings reported, ^18^F-PET/CT and functional imaging may be useful in the diagnosis and management of schistosomiasis: the role of ^18^F-FDG PET/CT in the definition of disease extension has been demonstrated, highlighting the sites of inflammation and infections difficult to detect by conventional morphological imaging [13]. Moreover, this imaging technique could be helpful in the eventual choice of biopsy sites: biopsy is performed usually in patients with a suspicious clinical picture for schistosomiasis and no eggs detected at microscopic examination of urine and stool [23]; the validity of ^18^F-FDG PET/CT in the selection of biopsy site has been reported in other inflammation and infections [81,82] and this imaging modality could be useful in schistosomiasis as well, since lesions are hypermetabolic on ^18^F-FDG PET/CT scan as suggested in the cases reported. In addition, some authors suggested the possible role of this imaging technique in treatment evaluation: further studies are needed, but this promising approach has been well-demonstrated in mice infected with *S. mansoni* and subsequently treated with praziquantel [64,83]. The main disadvantages of PET/CT are represented by the high cost of the procedure and the exposure to high radiation doses [84]. Moreover, it is important to underline that ^18^F-FDG PET/CT may present several limitations in the detection of cerebral schistosomiasis lesions due to the high physiological uptake of the radiopharmaceutical in the brain, thus hampering the visualization of possible focal hypermetabolic areas [85].

Beyond ^18^F-FDG, other PET tracers are commonly used in the evaluation of infections and inflammations: an example is represented by radiolabeled somatostatin analogues, radiopharmaceuticals that bind somatostatin receptors with high affinity [86,87,88]. Theoretically, we may suppose the applicability of these PET tracers in the assessment of inflammatory status induced by schistosome infection (it is well-demonstrated that somatostatin receptors are expressed by inflammatory cells) [89]. Nevertheless, it is important to mention the case reported by Marin-Martinez et al. in which a schistosomiasis lesion, localized in the retroperitoneum, showed no uptake of the radiolabeled somatostatin analogue: more evidence is needed to better understand these aspects [90].

Fibrosis markers are needed in the evaluation of schistosomiasis: in this context, an interesting PET radiopharmaceutical may be represented by ^68^Ga-labeled fibroblast activation protein inhibitor (^68^Ga-FAPI), a recent tracer that targets the fibroblast activation protein [91]; this radiopharmaceutical has been used to assess the degree of fibrosis in organs such as liver and kidneys [91,92] and potentially it may be useful for the assessment of fibrosis induced by schistosomiasis.

## 6. Conclusions

In this review, we have highlighted the meaningful role of diagnostic imaging techniques in the diagnosis of schistosomiasis. Radiology and nuclear medicine physicians should be aware of the possible characteristics of this disease, especially in case of migrants or travelers from endemic regions; in fact, in the last years non endemic countries have experienced significant flows of migrants from endemic areas and therefore, it is not uncommon to face this disease in daily practice. As we have underlined, epidemiological, clinical, and laboratory data are fundamental in the diagnosis of schistosomiasis, and diagnostic imaging techniques such as X-rays, US, CT, MRI, and PET/CT may be extremely helpful in the evaluation of the disease, also assessing the possible complications of schistosomiasis.

## Data Availability

All figures of the review are available from the corresponding author (A.Cimini) on request.
